# On Selection Criteria for the Tuning Parameter in Robust Divergence

**DOI:** 10.3390/e23091147

**Published:** 2021-09-01

**Authors:** Shonosuke Sugasawa, Shouto Yonekura

**Affiliations:** 1 Center for Spatial Information Science, The University of Tokyo, Chiba 277-8568, Japan; 2 Nospare Inc., Tokyo 107-0061, Japan; s.yonekura@chiba-u.jp; 3 Graduate School of Social Sciences, Chiba University, Chiba 263-8522, Japan

**Keywords:** efficiency, Hyvarinen score, outlier, unnormalized model

## Abstract

Although robust divergence, such as density power divergence and γ-divergence, is helpful for robust statistical inference in the presence of outliers, the tuning parameter that controls the degree of robustness is chosen in a rule-of-thumb, which may lead to an inefficient inference. We here propose a selection criterion based on an asymptotic approximation of the Hyvarinen score applied to an unnormalized model defined by robust divergence. The proposed selection criterion only requires first and second-order partial derivatives of an assumed density function with respect to observations, which can be easily computed regardless of the number of parameters. We demonstrate the usefulness of the proposed method via numerical studies using normal distributions and regularized linear regression.

## 1. Introduction

Data with outliers naturally arise in diverse areas. In the analysis of data containing outliers, statistical models with robust divergence are known to be powerful and have been used regularly. In particular, the density power divergence [1] and γ-divergence [2] have been routinely used in this context due to their robustness properties while there now exist others. In these studies, the theoretical properties of the robustness of robust divergence against outliers are also clarified through the analysis of influence functions. For its interesting applications, see for example [3,4] and references therein. Robust divergence, in general, holds a tuning parameter that controls robustness under model misspecification or contamination. Ref. [1] noted that there is a trade-off between estimation efficiency and strength of robustness; thereby, a suitable choice of the tuning parameter seems crucial in practice. However, a well-known selection strategy such as cross-validation is not straightforward under contamination, so that we need to rely on a trial-and-error way to find a reasonable value of the tuning parameter.

To select a turning parameter, we here propose a simple but novel selection criterion for the tuning parameter by using the asymptotic approximation of Hyvarinen score [5,6] with unnormalized models based on robust divergence. Typical existing methods [7,8] choose a tuning parameter based on the asymptotic approximation of the mean square error but have the drawback of requiring some pilot estimators and an analytical expression of the asymptotic variance. In addition, their works are essentially limited to the simple normal distribution and simple linear regression. Our proposed method has the following advantages over the existing studies.

1. Our method does not require an explicit representation of the asymptotic variance. Therefore, our method can be applied to rather complex statistical models, such as multivariate models, which seems difficult to be handled by the previous methods;
2. In the existing studies, it is necessary to determine a certain value as a pilot estimate to optimize a tuning parameter. Thus, the estimates may strongly depend on the pilot estimate. On the other hand, our method does not require a pilot estimate and is stable and statistically efficient;
3. Although our proposed method is based on a simple asymptotic expansion, it is more statistically meaningful and easier to interpret the results statistically than existing methods because it is based on the theory of parameter estimation for unnormalized statistical models.

Through numerical studies under simple settings, we show that the existing methods can be sensitive to a pilot estimate and tends to select an unnecessarily larger value of a tuning parameter, leading to loss of efficiency compared with the proposed method. Moreover, we still apply the proposed selection method, an estimation procedure in which the asymptotic variance is difficult to compute. As an illustrative example of such a case, we consider robust linear regression with γ-divergence and $\ell_{1}$-regularization, where the existing approach is infeasible to apply.

As related works, there are two information criteria using the Hyvarinen score. [9] proposed AIC-type information criteria for unnormalized models by deriving an asymptotic unbiased estimator of the Hyvarinen score, but it does not allow unnormalized models whose normalizing constants do not exist. Hence, the criterion cannot be applied to the current situation. On the other hand, [10] proposed an information criterion via Laplace approximation of the marginal likelihood in which the potential function is constructed by the Hyvarinen score. Although [10] covers unnormalized models with possibly diverging normalizing constants, the estimator used in the criterion is entirely different from one defined as the maximizer of robust divergence; thereby, the criterion does not apply to the tuning parameter selection of robust divergence either. Moreover, ref. [11] developed an robust sequential Monte Carlo sampler based on robust divergence in which γ is adaptively selected. However, it does not provide selection of γ in a frequentist framework.

The rest of the paper is organized as follows. Section 2 introduces a new selection criterion based on the Hyvarinen score. We then provide concrete expressions of the proposed criterion under density power divergence and γ-divergence in Section 3. We numerically illustrate the proposed method in two situations in Section 4. Concluding remarks are given in Section 5.

## 2. Tuning Parameter Selection of Robust Divergence

Suppose we observe $y_{1},\ldots ,y_{n}$ as realizations from a true distribution or data generating process *G*, and we want to fit a statistical model $\left\{f_{\theta }:\theta \in \Theta \right\}$ where $\Theta \subseteq R^{d}$ for some d≥1. Furthermore, assume that the density of *G* is expressed as $\left(1-\omega \right)f_{\theta^{*}}+\omega \delta$, where δ is a contaminated distribution that produces outliers in observations. Our goal is to make statistical inference on $\theta^{*}$ by successfully eliminating information of outliers. To this end, robust divergence such as density power divergence [1] and γ-divergence [2] is typically used for robust inference on $\theta^{*}$. Let $y=(y_{1},\ldots ,y_{n})$ be a vector of observations and $D_{\gamma }\left(y;\theta \right)$ be a (negative) robust divergence with a tuning parameter γ. We assume that the robust divergence has a additive form, namely, $D_{\gamma }\left(y;\theta \right)=\sum_{i=1}^{n}D_{\gamma }\left(y_{i};\theta \right)$. This constraint is necessary when using the H-score, but it is not a strong constraint in the context of this study because the well-known robust divergence, as presented in Section 3, satisfies this property.

For selecting the tuning parameter γ, our main idea is to regard $L_{\gamma }\left(y_{i};\theta \right)\equiv \exp\left\{D_{\gamma }\left(y_{i};\theta \right)\right\}$ as an unnormalized statistical model whose normalizing constant may not exist. Recently, ref. [10] pointed out that the role of such unnormalized models can be recognized in terms of relative probability. For such model, we employ the Hyvarinen score (H-score) in terms of Bayesian model selection [5,6], defined as

(1) $$H_{n}^{*}\left(\gamma \right)\equiv \frac{1}{n}\sum_{i=1}^{n}\left\{2\frac{\partial^{2}}{\partial y_{i}^{2}}\log L_{\gamma }^{\left(m\right)}\left(y\right)+{\left(\frac{\partial }{\partial y_{i}}\log L_{\gamma }^{\left(m\right)}\left(y\right)\right)}^{2}\right\},$$

where $L_{\gamma }^{\left(m\right)}\left(y\right)$ is the marginal likelihood given by

(2) $$L_{\gamma }^{\left(m\right)}\left(y\right)=\int \pi \left(\theta \right)\prod_{i=1}^{n}L_{\gamma }\left(y_{i};\theta \right)d\theta .$$

with some prior distribution π(θ). We consider an asymptotic approximation of the H-score (1) under large sample sizes. Under regularity conditions [12], the Laplace approximation of (2) is

(3) $$L_{\gamma }^{\left(m\right)}\left(y\right)\approx {\left(2\pi \right)}^{d/2}\pi \left({\hat{\theta }}_{\gamma }\right){\left|H\left({\hat{\theta }}_{\gamma }\right)\right|}^{-1/2}\prod_{i=1}^{n}L_{\gamma }\left(y_{i};{\hat{\theta }}_{\gamma }\right),$$

where ${\hat{\theta }}_{\gamma }$ is the M-estimator given by

$${\hat{\theta }}_{\gamma }={\mathrm{argmax}}_{\theta }\sum_{i=1}^{n}\log L_{\gamma }\left(y_{i};\theta \right),$$

and $H({\hat{\theta }}_{\gamma })$ is the Hessian matrix at $\theta ={\hat{\theta }}_{\gamma }$. Then, we have the following approximation, where the proof is deferred to Appendix A.

**Proposition** **1.** *Under some regularity conditions, it follows that*

$$\begin{array}{c} \frac{\partial }{\partial y_{i}}\log L_{\gamma }^{\left(m\right)}\left(y\right)=D_{\gamma }^{\prime }\left(y_{i};{\hat{\theta }}_{\gamma }\right)+o_{p}\left(1\right),\;\;\;\;\frac{\partial^{2}}{\partial y_{i}^{2}}\log L_{\gamma }^{\left(m\right)}\left(y\right)=D_{\gamma }^{\prime \prime }\left(y_{i};{\hat{\theta }}_{\gamma }\right)+o_{p}\left(1\right), \end{array}$$

*where $D_{\gamma }^{\prime }\left(y_{i};\theta \right)=\partial D_{\gamma }\left(y_{i};\theta \right)/\partial y_{i}$ and $D_{\gamma }^{\prime \prime }\left(y_{i};\theta \right)=\partial^{2}D_{\gamma }\left(y_{i};\theta \right)/\partial y_{i}^{2}$.*

The above results give the following approximation of the original H-score:

(4) $$H_{n}\left(\gamma \right)=\frac{1}{n}\sum_{i=1}^{n}\left\{2D_{\gamma }^{\prime \prime }\left(y_{i};{\hat{\theta }}_{\gamma }\right)+{\left(D_{\gamma }^{\prime }\left(y_{i};{\hat{\theta }}_{\gamma }\right)\right)}^{2}\right\},$$

which satisfies $H_{n}\left(\gamma \right)=H_{n}^{*}\left(\gamma \right)+o_{p}\left(1\right)$ under n→∞. We then define the optimal γ as

$$\gamma_{opt}={\mathrm{argmin}}_{\gamma }H_{n}\left(\gamma \right).$$

Let $\theta_{\gamma }^{*}$ be the quantity that ${\hat{\theta }}_{\gamma }$ converges to. Then, from the argument given in [5,10], the criterion (4) would converge to the Fisher divergence between the unnormalized model $\exp\{D_{\gamma }\left(y;\theta_{\gamma }^{*}\right)\}$ and the true data generating process. Although the unnormalized model is not a valid statistical model in the sense that it does not have a finite normalizing constant, the Fisher divergence can be recognized as the distance in terms of relative probabilities [10].

Existing selection strategies for γ mostly use the asymptotic variance of ${\hat{\theta }}_{\gamma }$. For example, under the density power divergence, refs. [7,8] suggested using asymptotic approximation of the mean squared errors of ${\hat{\theta }}_{\gamma }$. However, computation of the asymptotic variance is not straightforward, especially when an additional penalty function is incorporated into the objective function or the dimension of θ is large. On the other hand, the proposed criterion (4) does not require the computation of asymptotic variance but only needs the derivatives of robust divergence concerning $y_{i}$. Furthermore, it should be noted that the proposed criterion (4) can be applied to a variety of robust divergence.

## 3. Possible Robust Divergences to Consider

We here provide detailed expressions for the proposed criterion (4) under some robust divergences. For simplicity, we focus on two robust divergences which can be empirically estimated from the data. Still, the proposed method could be applied to other divergences such as Hellinger divergence [13] or αβ-divergence [14]. In what follows, we shall use the notations, $f^{\prime }\left(y_{i};\theta \right)=\partial f\left(y_{i};\theta \right)/\partial y_{i}$ and $f^{\prime \prime }\left(y_{i};\theta \right)=\partial^{2}f\left(y_{i};\theta \right)/\partial y_{i}^{2}$.

### 3.1. Density Power Divergence

The density power divergence [1] for a statistical model $f(y_{i};\theta )$ is

$$D_{\gamma }\left(y_{i};\theta \right)=\frac{1}{\gamma }f{\left(y_{i};\theta \right)}^{\gamma }-\frac{1}{1+\gamma }\int f{\left(t;\theta \right)}^{1+\gamma }dt.$$

It can be seen that $D_{\gamma }\left(y_{i};\theta \right)+1-1/\gamma \to \log f\left(y_{i};\theta \right)$ as γ→0, so the above function can be regarded as an extension of the standard log-likelihood. Then, a straightforward calculation leads to the expression of (4), given by

$$\begin{array}{cc} H_{n}\left(\gamma \right)=\sum_{i=1}^{n} & \left[f^{\prime }{\left(y_{i};{\hat{\theta }}_{\gamma }\right)}^{2}f{\left(y_{i};{\hat{\theta }}_{\gamma }\right)}^{\gamma -2}\left\{2\left(\gamma -1\right)+f{\left(y_{i};{\hat{\theta }}_{\gamma }\right)}^{\gamma }\right\}+2f{\left(y_{i};{\hat{\theta }}_{\gamma }\right)}^{\gamma -1}f^{\prime \prime }\left(y_{i};{\hat{\theta }}_{\gamma }\right)\right]. \end{array}$$

### 3.2. γ-Divergence

The original form of γ-divergence [2] for a statistical model $f(y_{i};\theta )$ is given by

$$\frac{1}{\gamma }\log\left\{\sum_{i=1}^{n}f{\left(y_{i};\theta \right)}^{\gamma }{\left(\int f{\left(t;\theta \right)}^{1+\gamma }dt\right)}^{-\gamma /(1+\gamma )}\right\},$$

which is not an additive form. However, the maximization of the above function with respect to θ is equivalent to the maximization of the transformed version of γ-divergence, $D_{\gamma }\left(y;\theta \right)=\sum_{i=1}^{n}D_{\gamma }\left(y_{i};\theta \right)$, where

$$D_{\gamma }\left(y_{i};\theta \right)=\frac{1}{\gamma }f{\left(y_{i};\theta \right)}^{\gamma }{\left\{\int f{\left(t;\theta \right)}^{1+\gamma }dt\right\}}^{-\gamma /(1+\gamma )}.$$

Then, we have

$$\begin{array}{cc} H_{n}\left(\gamma \right)=\sum_{i=1}^{n} & \left[f^{\prime }{\left(y_{i};{\hat{\theta }}_{\gamma }\right)}^{2}f{\left(y_{i};{\hat{\theta }}_{\gamma }\right)}^{\gamma -2}\left\{\frac{2(\gamma -1)}{C_{\gamma }\left({\hat{\theta }}_{\gamma }\right)}+\frac{f{\left(y_{i};{\hat{\theta }}_{\gamma }\right)}^{\gamma }}{C_{\gamma }{\left({\hat{\theta }}_{\gamma }\right)}^{2}}\right\}+\frac{2f{\left(y_{i};{\hat{\theta }}_{\gamma }\right)}^{\gamma -1}f^{\prime \prime }\left(y_{i};{\hat{\theta }}_{\gamma }\right)}{C_{\gamma }\left({\hat{\theta }}_{\gamma }\right)}\right], \end{array}$$

where $C_{\gamma }\left(\theta \right)={\left(\int f{\left(t;\theta \right)}^{1+\gamma }dt\right)}^{\gamma /(1+\gamma )}$.

## 4. Numerical Examples

### 4.1. Normal Distribution with Density Power Divergence

We first consider a simple example of robust estimation of the normal population mean under unknown variance. Let $y_{1},\ldots ,y_{n}$ be sampled observations and we fit $N(\mu ,\sigma^{2})$ to the data. The density power divergence [1] of the model is given by

$$D_{\gamma }\left(y_{i};\mu ,\sigma^{2}\right)=\frac{1}{\gamma }\phi {\left(y_{i};\mu ,\sigma^{2}\right)}^{\gamma }-{\left(2\pi \sigma^{2}\right)}^{-\gamma /2}{\left(1+\gamma \right)}^{-3/2},$$

where $\phi (y_{i};\mu ,\sigma^{2})$ is the density function of $N(\mu ,\sigma^{2})$. In this case, the criterion (4) is expressed as

$$H_{n}\left(\gamma \right)=\sum_{i=1}^{n}\left[\frac{2\left\{\gamma {\left(y_{i}-{\hat{\mu }}_{\gamma }\right)}^{2}-{\hat{\sigma }}_{\gamma }^{2}\right\}}{{\hat{\sigma }}_{\gamma }^{4}}\phi {\left(y_{i};{\hat{\mu }}_{\gamma },{\hat{\sigma }}_{\gamma }^{2}\right)}^{\gamma }+\frac{{\left(y_{i}-{\hat{\mu }}_{\gamma }\right)}^{2}}{{\hat{\sigma }}_{\gamma }^{4}}\phi {\left(y_{i};{\hat{\mu }}_{\gamma },{\hat{\sigma }}_{\gamma }^{2}\right)}^{2\gamma }\right],$$

where ${\hat{\mu }}_{\gamma }$ and ${\hat{\sigma }}_{\gamma }$ are the estimator based on the density power divergence.

We first demonstrate the proposed selection strategy through simulation studies. We simulated $y_{1},\ldots ,y_{n}$ from the normal distribution with true parameters, μ=2, and σ=1, and then replace the first nω observations by $y_{i}+7$. We adopted four settings for ω∈{0,0.05,0.1,0.15}. Using the simulated dataset, the optimal γ is selected among {0,0.01,…,0.69,0.70} through the criterion $H_{n}\left(\gamma \right)$, and we obtain the adaptive estimator ${\hat{\mu }}_{\gamma_{opt}}$. For comparison, we also employed two selection methods, OWJ [7] and IWJ [8], in which the optimal value of γ is selected via asymptotic approximation of mean squared errors of the estimator. We set γ=0.5 to compute a pilot estimator that must be specified in the two methods. Furthermore, we also computed ${\hat{\mu }}_{\gamma }$ with γ=0.1,0.3, and 0.5. Using an estimator of the asymptotic variance of ${\hat{\mu }}_{\gamma }$ given in [8], we also computed the Wald-type 95% confidence interval of μ. Based on 5000 simulated datasets, we obtained the squared root of mean squared error (RMSE) of the point estimator, as well as coverage probability (CP) and average length (AL) of the interval estimation. The results are reported in Table 1. It is observed that the use of small γ (such as γ=0.1) may lead to unsatisfactory results when the contamination is heavy. It can also be seen that with the use of relatively large γ, the estimation results can be inefficient. On the other hand, the proposed method can adaptively select a suitable value of γ since the averaged value of $\gamma_{opt}$ increases with the contamination ratio ω, as confirmed in Table 2. This would be the main reason that the proposed method provides reasonable performance in all the scenarios.

**Figure 1:**
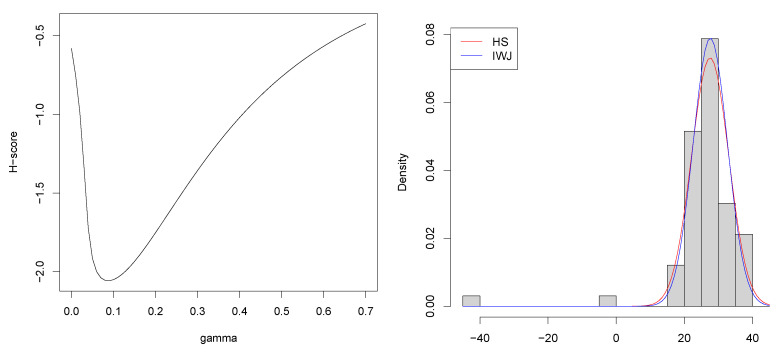
H-scores for each γ (**left**) and the estimate normal density functions with optimal gamma selected via the H-score and IJW methods (**right**) in the analysis of the Newcomb data.

We next apply the proposed method to Simon Newcomb’s measurements of the speed of light data, motivated by applications in Basu et al. [1], Basak et al. [8], Stigler [15]. We searched the optimal γ among {0,0.01,…,0.69,0.70} and the H-sores are shown in left panel in Figure 1. The obtained optimal value is $\gamma_{\mathrm{opt}}=0.09$, which is substantially smaller than $\hat{\gamma }=0.23$ selected by the existing methods as reported in [8]. Since the method proposed in [8] requires a pilot estimate and the estimation results depend significantly on it, we believe that our estimation results are more reasonable. In fact, it is unlikely that we will have to use a value of γ=0.23 for a dataset that contains only two outliers. As shown in the right panel in Figure 1, the estimated density functions are almost the same when γ=0.09 and when γ=0.23. However, it would be preferable to adopt the smaller value of γ=0.09 if the estimates are almost identical in terms of statistical efficiency.

### 4.2. Gamma Distribution with Density Power Divergence

We next consider robust estimation of the gamma distribution. Let $y_{1},\ldots ,y_{n}$ be sampled observations and we fit Ga(α,β) to the data, where α is a shape parameter and β is a rate parameter, so that the expectation of the gamma distribution is α/β. The density power divergence of the model is given by

$$D_{\gamma }\left(y_{i};\alpha ,\beta \right)=\frac{1}{\gamma }f_{\mathrm{Ga}}{\left(y_{i};\alpha ,\beta \right)}^{\gamma }-\frac{\Gamma (\alpha (1+\gamma )-\gamma )}{\Gamma {\left(\alpha \right)}^{1+\gamma }}\beta^{\gamma }{\left(1+\gamma \right)}^{-\alpha (1+\gamma )+\gamma },$$

where $f_{\mathrm{Ga}}\left(y_{i};\alpha ,\beta \right)$ is the density function of Ga(α,β). In this case, the criterion of γ is one given in Section 3.1 in which the first and second order derivatives of the density are given as

$$\begin{array}{cc} f_{\mathrm{Ga}}^{\prime }\left(y_{i};\alpha ,\beta \right) & =\left(\frac{\alpha -1}{y_{i}}-\beta \right)f_{\mathrm{Ga}}\left(y_{i};\alpha ,\beta \right)\\ f_{\mathrm{Ga}}^{\prime \prime }\left(y_{i};\alpha ,\beta \right) & =\left(\frac{\alpha -1}{y_{i}}-\beta \right)f_{\mathrm{Ga}}^{\prime }\left(y_{i};\alpha ,\beta \right)-\frac{\alpha -1}{y_{i}^{2}}f_{\mathrm{Ga}}\left(y_{i};\alpha ,\beta \right). \end{array}$$

We demonstrate the proposed selection criterion through simulation studies. We simulated $y_{1},\ldots ,y_{n}$ from the gamma distribution with true parameters, α=2, and β=4, and then replace the first nω observations by $y_{i}+5$. We adopted three settings for ω∈{0,0.05,0.1} and two scenarios for n∈{100,200}. Using the simulated dataset, the optimal γ is selected among {0,0.01,…,0.49,0.50} through the HS criterion $H_{n}\left(\gamma \right)$, and we obtain the adaptive estimators, ${\hat{\alpha }}_{\gamma_{opt}}$ and ${\hat{\beta }}_{\gamma_{opt}}$. For comparison, we applied the standard maximum likelihood (ML) estimator, as well as the robust estimator with fixed γ∈{0.1,0.5}. In this study, we do not include OWJ or IWJ methods since the asymptotic variance formula in this case has not been investigated before and the derivation would require tedious algebraic calculation.

Based on 5000 simulated datasets, we obtained the squared root of mean squared error (RMSE) of the point estimator, where the results are shown in Table 3. We also provide the average values of the selected γ in Table 4. It is observed that the (non-robust) ML and the robust method using the small γ (γ=0.1) leads to unsatisfactory results when the data are contaminated. It can also be confirmed that γ=0.5 does not hold reasonable accuracy when the contamination ratio is small or 0, which indicates that a suitable selection step is substantially related to the estimation result. The proposed method, however, has some adaptive property. When there is not contamination, the estimation performance is almost identical to the the ML estimator which is the most efficient in this scenario since a small value (sometimes exactly zero) of γ is selected in this scenario, as shown in Table 4. On the other hand, the estimation performance is still better than the other methods when the data are contaminated, by successfully finding a suitable value (increasing according to ω) of γ.

### 4.3. Regularized Linear Regression with γ-Divergence

Note that the proposed criterion can be used when some regularized terms are introduced in the objective function, while the existing method requiring an asymptotic variance of the estimator is not simply applicable. We demonstrate the advantage of the proposed method through regularized linear regression with γ-divergence [16]. Let $y_{i}$ and $x_{i}$ be a response variable and a *p*-dimensional vector of covariates, respectively, for i=1,…,n. The model is $y_{i}∼N\left(x_{i}^{⊤}\beta ,\sigma^{2}\right)$. Then, the transformed γ-divergence is $D_{\gamma }\left(y_{i};\theta \right)=\gamma^{-1}\phi {\left(y_{i};x_{i}^{⊤}\beta ,\sigma^{2}\right)}^{\gamma }/C_{\gamma }\left(\sigma^{2}\right)$ with $C_{\gamma }\left(\sigma^{2}\right)={\left\{{\left(1+\gamma \right)}^{-1/2}{\left(2\pi \sigma^{2}\right)}^{-\gamma /2}\right\}}^{\gamma /(1+\gamma )}$, and the H-score is expressed as

$$H_{n}\left(\gamma \right)=\sum_{i=1}^{n}\left[\frac{2\left\{\gamma {\left(y_{i}-x_{i}^{⊤}{\hat{\beta }}_{\gamma }\right)}^{2}-{\hat{\sigma }}_{\gamma }^{2}\right\}}{{\hat{\sigma }}_{\gamma }^{4}C_{\gamma }\left({\hat{\sigma }}_{\gamma }^{2}\right)}\phi {\left(y_{i};x_{i}^{⊤}{\hat{\beta }}_{\gamma },{\hat{\sigma }}_{\gamma }^{2}\right)}^{\gamma }+\frac{{\left(y_{i}-x_{i}^{⊤}{\hat{\beta }}_{\gamma }\right)}^{2}}{{\hat{\sigma }}_{\gamma }^{4}C_{\gamma }{\left({\hat{\sigma }}_{\gamma }^{2}\right)}^{2}}\phi {\left(y_{i};x_{i}^{⊤}{\hat{\beta }}_{\gamma },{\hat{\sigma }}_{\gamma }^{2}\right)}^{2\gamma }\right].$$

Here, ${\hat{\beta }}_{\gamma }$ and ${\hat{\sigma }}_{\gamma }^{2}$ are estimated as the minimizer of the following regularized γ-divergence:

$$-\frac{1}{\gamma }\log\left\{\sum_{i=1}^{n}\phi {\left(y_{i};x_{i}^{⊤}\beta ,\sigma^{2}\right)}^{\gamma }\right\}-\frac{\gamma }{1+\gamma }\log\sigma^{2}+\lambda \sum_{k=1}^{p}\left|\beta_{k}\right|,$$

where λ is an additional tuning parameter that can be optimized via 10-fold cross-validation. We use the R package gamreg [16] to estimate β and $\sigma^{2}$ under given γ.

**Figure 2:**
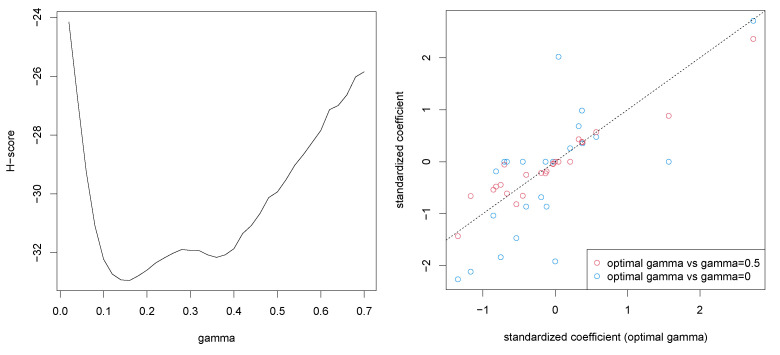
H-scores for each γ (**left**) and the estimated regression coefficients with three choices of γ (**right**).

We apply the aforementioned method to the well-known Boston housing dataset [17]. In this analysis, we included the original 13 covariates and 12 quadratic terms of the covariates except for one binary covariate, resulting in 25 covariates in total. We searched the optimal γ among {0,0.02,…,0.68,0.70}, and the estimated H-scores are shown in the left panel in Figure 2, where the optimal value of γ was 0.16. For comparison, we estimated the regression coefficients with γ=0 and γ=0.5. Note that γ=0 reduces to the (non-robust) standard regularized linear regression. The scatter plots of the estimated standardized coefficients under γ=0.16 against ones under the two choices of γ are shown in the right panel of Figure 2. It is confirmed that the estimates with γ=0.16 and γ=0.5 are comparable while there are substantial differences between estimates with γ=0.16 and γ=0, indicating that a certain amount of robustness is required for the dataset.

## 5. Concluding Remarks

We proposed a new criterion for selecting the optimal tuning parameter in robust divergence, using the Hyvarinen score for unnormalized models with robust divergence. The proposed criterion does not require the asymptotic variance formula of the estimator that is needed in the existing selection methods. Although we simply focused on the univariate and continuous situation, the proposed criterion can also be applied to multivariate or discrete distribution, where finite differences under discrete distributions should replace derivatives. Applications of the proposed score to such cases would also be helpful, and we left it to future work.

## Figures and Tables

**Table 1:** RMSE of the point estimation and CP and AL of interval estimation of the normal population mean.

					Fixed γ		
	ω	HS	OWJ	IWJ	0.1	0.3	0.5
	0	10.3	10.6	10.3	10.2	10.5	11.0
RMSE	0.05	10.7	10.9	10.7	14.4	10.8	11.3
	0.1	11.0	11.1	11.0	44.7	11.1	11.5
	0.15	11.4	11.4	11.4	82.6	11.5	11.8
	0	94.8	93.8	94.2	94.6	94.5	94.4
CP	0.05	94.7	93.9	94.1	93.2	94.2	94.1
	0.1	94.3	94.1	94.2	36.7	94.2	94.4
	0.15	94.1	93.7	93.8	0.1	93.6	94.1
	0	40.6	40.1	39.8	40.4	40.7	42.6
AL	0.05	41.7	41.0	40.9	50.4	41.2	43.3
	0.1	42.5	41.9	41.8	79.5	42.0	44.1
	0.15	43.4	42.9	42.9	100.4	43.1	45.1

**Table 2:** Average values of selected γ in the three methods in simulation studies with normal distribution.

ω	HS	OWJ	IWJ
0	0.088	0.212	0.158
0.05	0.169	0.260	0.230
0.1	0.217	0.284	0.267
0.15	0.252	0.302	0.294

**Table 3:** RMSE of the point estimation in the gamma distribution.

		α				β			
				Fixed γ				Fixed γ	
*n*	ω	ML	HS	0.1	0.5	ML	HS	0.1	0.5
	0	0.28	0.29	0.38	1.25	0.65	0.73	0.91	3.63
100	0.05	0.91	0.37	0.70	1.29	2.50	1.00	1.98	3.74
	0.1	1.13	0.40	0.99	1.34	3.10	1.09	2.81	3.86
	0	0.19	0.20	0.29	1.14	0.44	0.49	0.70	3.37
200	0.05	0.92	0.28	0.69	1.18	2.54	0.75	1.98	3.47
	0.1	1.14	0.28	1.01	1.21	3.13	0.78	2.86	3.53

**Table 4:** Average values of selected γ by the proposed criterion in the gamma distribution.

	n=100			n=200		
ω	0	0.05	0.1	0	0.05	0.1
$\gamma_{\mathrm{opt}}$	0.036	0.137	0.164	0.025	0.133	0.161

## Appendix A. Proof of Proposition 1

We first assume standard regularity conditions in the M-estimation theory [18] for the objective function $\sum_{i=1}^{m}\log L_{\gamma }\left(y_{i};\theta \right)$. We also assume that $\log L_{\gamma }\left(y_{i};\theta \right)$ is twice continuously differentiable with respect to $y_{i}$, logπ(θ) is continuously differentiable and the derivative of logπ(θ) is bounded.

We first note that ${\hat{\theta }}_{\gamma }$ is a solution of the following estimating equation:

$$\sum_{i=1}^{n}S_{\gamma }\left(y_{i};\theta \right)=0,\;\;\;\;\;\;S_{\gamma }\left(y_{i};\theta \right)\equiv \frac{\partial }{\partial \theta }\log L_{\gamma }\left(y_{i};\theta \right).$$

From the implicit function theorem, it follows that

$$\begin{array}{cc} \frac{\partial {\hat{\theta }}_{\gamma }}{\partial y_{i}}\; & ={\left\{\sum_{j=1}^{n}\frac{\partial }{\partial \theta }S_{\gamma }\left(y_{j};\theta \right){|}_{\theta ={\hat{\theta }}_{\gamma }}\right\}}^{-1}\frac{\partial }{\partial y_{i}}\sum_{j=1}^{n}S_{\gamma }\left(y_{j};\gamma \right){|}_{\theta ={\hat{\theta }}_{\gamma }}=H{\left({\hat{\theta }}_{\gamma }\right)}^{-1}S_{\gamma }^{\prime }\left(y_{i};{\hat{\theta }}_{\gamma }\right), \end{array}$$

where we defined $S_{\gamma }^{\prime }\left(y_{i};\theta \right)=\partial S_{\gamma }\left(y_{i};\theta \right)/\partial y_{i}$. Note that $\partial {\hat{\theta }}_{\gamma }/\partial y_{i}=O_{p}\left(n^{-1}\right)$ under large *n*. From (3), the first order partial derivative of the marginal log-likelihood can be approximated as

(A1) $$\frac{\partial }{\partial y_{i}}\log L_{\gamma }^{\left(m\right)}\left(y\right)\approx \frac{\partial }{\partial y_{i}}\sum_{j=1}^{n}\log L_{\gamma }\left(y_{j};{\hat{\theta }}_{\gamma }\right)+\frac{\partial }{\partial y_{i}}\log\pi \left({\hat{\theta }}_{\gamma }\right)-\frac{1}{2}\frac{\partial }{\partial y_{i}}\log\left|H\left({\hat{\theta }}_{\gamma }\right)\right|.$$

Under the regularity conditions for π(θ), it follows that

$$\frac{\partial }{\partial y_{i}}\log\pi \left({\hat{\theta }}_{\gamma }\right)=\frac{\partial {\hat{\theta }}_{\gamma }}{\partial y_{i}}\times \frac{\partial }{\partial \theta }\log\pi \left(\theta \right){|}_{\theta ={\hat{\theta }}_{\gamma }}=o_{p}\left(1\right)$$

under large *n*. From the same argument, we can also show that $\partial \log|H\left({\hat{\theta }}_{\gamma }\right)|/\partial y_{i}=o_{p}\left(1\right)$. Regarding the first term in (A1), we have

(A2) $$\begin{array}{cc} \frac{\partial }{\partial y_{i}}\sum_{j=1}^{n}\log L_{\gamma }\left(y_{j};{\hat{\theta }}_{\gamma }\right) & =\frac{\partial }{\partial y_{i}}\log L_{\gamma }\left(y_{i};\theta \right){|}_{\theta ={\hat{\theta }}_{\gamma }}+{\left(\frac{\partial {\hat{\theta }}_{\gamma }}{\partial y_{i}}\right)}^{⊤}\sum_{j=1}^{n}\frac{\partial }{\partial \theta }\log L_{\gamma }\left(y_{j};\theta \right){|}_{\theta ={\hat{\theta }}_{\gamma }}\\ & =D_{\gamma }^{\prime }\left(y_{i};{\hat{\theta }}_{\gamma }\right)+{\left\{\sum_{j=1}^{n}S_{\gamma }\left(y_{j};{\hat{\theta }}_{\gamma }\right)\right\}}^{⊤}H{\left({\hat{\theta }}_{\gamma }\right)}^{-1}S_{\gamma }^{\prime }\left(y_{i};{\hat{\theta }}_{\gamma }\right)\\ & =D_{\gamma }^{\prime }\left(y_{i};{\hat{\theta }}_{\gamma }\right)+O_{p}\left(n^{-1/2}\right), \end{array}$$

since $S_{\gamma }\left(y_{j};\theta \right)$ is a score function and $\sum_{j=1}^{n}S_{\gamma }\left(y_{j};{\hat{\theta }}_{\gamma }\right)=O_{p}\left(n^{1/2}\right)$.

Using the expression of the first order derivative (A2), it holds that

(A3) $$\begin{array}{cc} \frac{\partial^{2}}{\partial y_{i}^{2}}\sum_{j=1}^{n}\log L_{\gamma }\left(y_{j};{\hat{\theta }}_{\gamma }\right) & =\frac{\partial }{\partial y_{i}}D_{\gamma }^{\prime }\left(y_{i};{\hat{\theta }}_{\gamma }\right)+{\left\{\frac{\partial }{\partial y_{i}}\sum_{j=1}^{n}S_{\gamma }\left(y_{j};{\hat{\theta }}_{\gamma }\right)\right\}}^{⊤}H{\left({\hat{\theta }}_{\gamma }\right)}^{-1}S_{\gamma }^{\prime }\left(y_{i};{\hat{\theta }}_{\gamma }\right)\\ & +{\left\{\sum_{j=1}^{n}S_{\gamma }\left(y_{j};{\hat{\theta }}_{\gamma }\right)\right\}}^{⊤}H{\left({\hat{\theta }}_{\gamma }\right)}^{-1}\left\{\frac{\partial }{\partial y_{i}}H\left({\hat{\theta }}_{\gamma }\right)\right\}H{\left({\hat{\theta }}_{\gamma }\right)}^{-1}S_{\gamma }^{\prime }\left(y_{i};{\hat{\theta }}_{\gamma }\right)\\ & +{\left\{\sum_{j=1}^{n}S_{\gamma }\left(y_{j};{\hat{\theta }}_{\gamma }\right)\right\}}^{⊤}H{\left({\hat{\theta }}_{\gamma }\right)}^{-1}\frac{\partial }{\partial y_{i}}S_{\gamma }^{\prime }\left(y_{i};{\hat{\theta }}_{\gamma }\right). \end{array}$$

Note that

$$\frac{\partial }{\partial y_{i}}D_{\gamma }^{\prime }\left(y_{i};{\hat{\theta }}_{\gamma }\right)=D_{\gamma }^{\prime \prime }\left(y_{i};{\hat{\theta }}_{\gamma }\right)+{\left(\frac{\partial }{\partial \theta }D^{\prime }\left(y_{i};\theta \right){|}_{\theta ={\hat{\theta }}_{\gamma }}\right)}^{⊤}\frac{\partial {\hat{\theta }}_{\gamma }}{\partial y_{i}}=D_{\gamma }^{\prime \prime }\left(y_{i};{\hat{\theta }}_{\gamma }\right)+O_{p}\left(n^{-1}\right).$$

By applying the same formula to $\partial S_{\gamma }^{\prime }\left(y_{i};{\hat{\theta }}_{\gamma }\right)/\partial y_{i}$, we can confirm that the third and forth terms in (A3) are $O_{p}\left(n^{-1/2}\right)$. Regarding the second term in (A3), we have

$$\begin{array}{cc} \frac{\partial }{\partial y_{i}}\sum_{j=1}^{n}S_{\gamma }\left(y_{j};{\hat{\theta }}_{\gamma }\right) & =S_{\gamma }^{\prime }\left(y_{i};{\hat{\theta }}_{\gamma }\right)+\left\{\sum_{j=1}^{n}\frac{\partial }{\partial \theta }S_{\gamma }\left(y_{j};\theta \right){|}_{\theta ={\hat{\theta }}_{\gamma }}\right\}H{\left({\hat{\theta }}_{\gamma }\right)}^{-1}S_{\gamma }^{\prime }\left(y_{i};{\hat{\theta }}_{\gamma }\right)\\ & =2S_{\gamma }^{\prime }\left(y_{i};{\hat{\theta }}_{\gamma }\right), \end{array}$$

which shows that the second term in (A3) is $O_{p}\left(n^{-1}\right)$, so that the proof is completed.
